# 基于标准化术语的胸外科结构化电子病历实践

**DOI:** 10.3779/j.issn.1009-3419.2018.04.03

**Published:** 2018-04-20

**Authors:** 杰 姜, 修义 于

**Affiliations:** 361003 厦门，厦门大学附属第一医院 The First Affiliated Hospital of Xiamen University, Xiamen 361003, China

**Keywords:** 结构化电子病历, 胸外科电子病历, 标准化诊疗术语, Structured electronic medical record, Thoracic surgery electronic medical record, Standard medical vocabulary

## Abstract

电子病历作为现代化医院信息化建设中的重要载体，越来越往精细化、智能化方向发展。本文就胸外科标准化、结构化电子病历系统进行介绍，并提出效果评价和展望。

## Abstract (en)

As an important carrier in the information construction of modern hospitals, electronic medical record is becoming more and more refined and intelligent. This paper introduces the standardized and structured electronic medical record system of thoracic surgecal department and puts forward the effect evaluation and prospect.

## 1 引言

电子病历从2007年开始提出，经过不断发展，已经成为目前医院信息化建设的核心内容^[1]^，电子病历的目标和意义并不在于要取代纸张病历，而是应具备三个内涵：一是包含了患者的完整信息并能进行共享；二是能提供医疗提示和报警；三是能提供资料库支持^[2]^。要实现这三个内涵，标准化和结构化是根本^[3]^。

许多专家认为在电子病历逐步迈入深度应用的阶段，标准化和结构化问题将成为制约电子病历实现区域共享、驱动职能决策、开展科研统计分析的最大障碍。美国宾夕法尼亚大学医疗体系在相应的调研报告中就有27种记录性别的方式。这种缺乏标注方式一致性的情形，严重阻碍了人们在数据库中进行信息检索^[3]^。国家对于电子病历的框架结构以及技术标准都没有统一规范，任由一些开发商自主进行，导致各种信息之间由于受到一定的阻碍而交流不畅，许多医院在建设的过程中，简单的将纸质病历转化为电子病历，文本病历大量存在，不能使得电子病历的优势完全发挥出来。

近年来，国家卫计委统计信息中心先后制修订了电子病历基本数据集等150余项卫生信息标准^[4]^。同时也颁布了《电子病历基本架构与数据标准（试行）》，对于电子病历的结构化提出框架性要求。但是对于专科的电子病历没有成立相应的标准和规范。经文献检索，笔者也尚未找到国内胸外科的规范化电子病历报道，医院胸外科电子病历存在着术语不标准、不统一的问题，例如" 食道癌" 和" 食管癌" 同时在临床上使用，并且也存在着文本病历大量存在，无法进行有效的临床科研数据统计分析，临床决策等问题。

中国医师协会胸外科分会胸外科专业术语标准化委员会在2017年推出我国首部《胸外科疾病标准化诊疗术语》，从顶层设计上解决胸外科临床诊疗术语标准化问题，厦门大学附属第一医院以此为契机，将标准化诊疗术语维护进入电子病历系统，并进行大量结构化、个性化数据节点设计，实现自动关联，互通共享，取得良好成效。

## 2 标准化诊疗术语概念

标准化诊疗术语就是在临床诊疗过程中所用到的基本语言进行统一命名，严格定义，选择或确立最恰当的术语，以解决临床所使用的术语异名繁多、或一词多义，同名异义，或异名同义等不规范、不统一的现象。

## 3 标准化电子病历概念

标准化电子病历是指术语标准化之上更高层次的信息模型的标准化，是在特定语用环境下的信息模型标准。

## 4 结构化电子病历概念

结构化电子病历是指从医学信息学角度，将以自然语言方式录入的医疗文书按照医学术语要求进行结构化分析，并将这些语义结构最终以关系型（面向对象）结构的方式保存到数据库中。

## 5 胸外科结构化电子病历的产生

厦门大学附属第一医院胸外科在《电子病历基本架构与数据标准》基础上开展了具有特色结构化、标准化电子病历模板。首先在病历术语使用上，完全依照《胸外科疾病标准化诊疗术语》并根据临床和科研需要进行扩充。采用互联互通、自动采集的形式写在病历上，能减轻医生的工作负担，缩短患者时间。在教学科研以及智能决策需求上，用自然语言写病历，" 结构化" 为日后查询和管理以及大数据的应用提供了基础。

根据《胸外科标准化诊疗术语》，胸外科结构化的病历模版有：入院记录、首次病程记录、手术相关记录、出院记录和病案首页。并在系统设计相应的核心表单。详见图 1。

**1:**
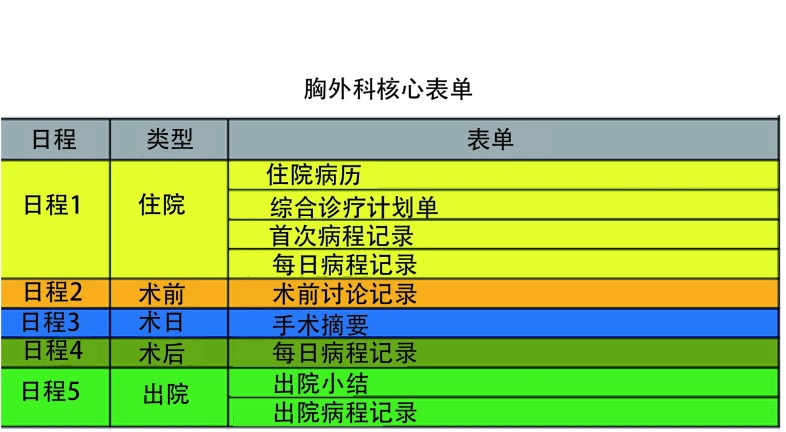
胸外科核心表单 The core forms of the thoracic surgecal department

## 6 胸外科结构化病历运用情况

### 6.1 入院记录

#### 6.1.1

在入院病历书写时，设计主诉点击勾选症状（支持拼音快速搜索）。

#### 6.1.2

在现病史一栏维护相应的专病模板：胸部外伤、食管癌、手汗症、胸腺瘤伴重症肌无力、肺癌—新辅助化疗后、肺癌、食管癌—化疗、肺占位性病变、纵膈肿瘤、自发性气胸，点击专病模板对于伴随症状进行点选。

#### 6.1.3

在家族史中，如选择有家族史，可扩展记录家属的关系、肿瘤的名称等信息。

#### 6.1.4

设计胸外科专科检查表单，主要有视诊、触诊、叩诊、听诊四大项目，默认正常查体结果，可通过点击相应栏目，打开下拉菜单进行阳性体征选择。

#### 6.1.5 诊断智能推荐

填写初步诊断时，根据所输入的主诉、现病史、既往史，通过内置知识库，推荐可能的诊断选项。

#### 6.1.6

选取诊断后，系统自动推送相应临床路径的提示，通过点击快速进入临床路径进行医嘱的选择。

#### 6.1.7 综合诊疗计划单

根据入院记录所选择的初步诊断，自动推荐诊疗方案，并自动关联该诊疗方案的预期目标。可通过拓展知识库增加诊疗方案。

### 6.2 首次病程记录

根据之前确定的初步诊断及诊疗计划，于新建首次病程记录时，自动生成相应鉴别诊断及术前检查项目。若初步诊断变更，则首次病程记录相应的鉴别诊断、诊疗计划、术前检查项目自动变更。

### 6.3 每日病程记录

新建日常病程记录时，自动获取昨日8时到今日8时24 h内新出数据，包括：未完成的检查项目、有异常结果的检验报告、新出的检查报告、新的会诊意见。在患者反馈栏中可点击患者不适反应进行记录，并激活“诊疗方案变更栏”，和相应的临床路径提示。

### 6.4 术前讨论

#### 6.4.1 术前功能评估

在术前功能评估区块，通过右键点击相应评估项目，进行相应功能分级的选择，其中评估的项目有：全身状况、肺功能、动脉血气分析、心功能、肝功能、肾功能和凝血功能评估。

#### 6.4.2 术前病情评估

在术前病情评估中，设计了肺癌、肺占位性病变、食管癌、纵膈肿瘤、自发性气胸、手汗症专病模板，点击选择不同内容的评估。例如肺癌病情评估项目主要有：肿瘤大小、计算机断层扫描（computed tomography, CT）值、外侵范围、同侧肺转移灶、阻塞性肺炎、转移淋巴、远处转移淋巴、临床分期等。通过右键点击相应评估栏目，在弹窗内点选评估的结果。

#### 6.4.3

在手术指征区块，通过选择模板并进行针对性评估，例如肺手术指征评估有手术适应症和手术绝对禁忌症、手术相对禁忌症。手术绝对禁忌症内容分别为：远处转移、对侧肺门、纵膈淋巴结转移、胸腔内器官广泛受侵、严重心肺、肝肾功不全、合并出血性疾病；手术相对禁忌症中有隆突增宽固定、喉返神经麻痹、膈神经麻痹、肺功能损害、伴有其他器官功能损害。根据点选内容依次排除手术禁忌症，确保每台手术的合理性。

#### 6.4.4 术前小结

完成术前讨论记录后，新建术前小结可自动获取相关记录。

### 6.5 手术摘要

设计术后结构化记录表单，根据专病模板选择相应的数据节点，主要记录节点有：手术方式、术中所见、手术并发症、手术总时长、留置管道、术中失血量、输血、有无植入物、快速冰冻等，这些数据节点均可扩展，例如手术方式选择胸部手术方式，二次扩展为肺部小结节定位技术、电视胸腔镜手术、开胸手术、机器人胸外科手术（预留）、中转开胸手术。其中电视胸腔镜手术又扩展为：单孔、两孔、三孔、四孔胸腔镜手术；根据专病模板，选择术中所见重要信息，例如肺癌根治术中结构化节点有：胸腔内粘连、胸腔积液、胸腔内其他占位、肿瘤定位（内置节点：左上叶、左下叶、右上叶、右中叶、右下叶）、肿瘤最大径、肿瘤表面胸膜凹陷、肿瘤外侵（内置节点累及范围：相邻肺叶、叶支气管、主支气管、胸壁等）、手术切除范围、淋巴结清扫情况（左侧：5、6、7、9、10L、11；右侧：2R、4R、7、9、10R、11）、预计转移性淋巴结、手术并发症（例有：呼吸系统并发症—持续性漏气、气胸、手术后胸腔积液等）、手术总时长、术中失血量、术后诊断、术后处理措施（自动获取）等。

### 6.6 术后病程记录

新建日常病程记录时，自动记录术后天数（用于标记术后事件的时间点），除上述增加每日病程记录基本点外，增加伤口情况、引流情况、手术并发症、治疗措施、诊疗方案等数据采集点。例如记录引流情况，记录引流情况，可手动增加多条引流管记录，包含引流管位置、通畅程度、颜色、性状、引流量、有无漏气、夹闭情况等。

### 6.7 出院小结

#### 6.7.1

在出院小结" 病理诊断" 区块，设计标本位置、分化程度、上皮来源、病理分期、有无基因测序数据节点，其中" 病理分期" 节点选择所有符合病理报告的分期记录，T/N分期细则分解为独立节点，系统自动识别最高分期，并根据最新版指南自动计算病理分期。

#### 6.7.2

在出院小结" 主要治疗经过" 区块，自动获取手术时间、手术方式、术后治疗措施。若患者未手术，可选择“非手术病人”，记录未手术的原因。

#### 6.7.3

在出院小结" 出院医嘱”区块，可选择出院医嘱。为下一步患者随访管理做准备。

### 6.8 出院病程记录

新建出院病程记录可自动获取的项目包括：手术到出院的天数、病理诊断、病理分期、基因检测结果、手术并发症、出院诊断、出院医嘱。

## 7 胸外科结构化、标准化电子病历的特点

胸外科电子病历在设计和实际运用上面，具有自身的特色：①根据《胸外科疾病标准化诊疗术语标准》，结合胸外科实际诊疗和科研统计的要求，构建胸外科电子病历标准化诊疗术语标准库，存储在CDR上面供调用。②通过接口实现外部系统病历生成和数据利用。在电子病历中可自动获取院内或其他关联结构的检验检查结果、医嘱、院前用药、会诊记录等信息。③实现模板关联病种、性别，可根据门诊诊断或患者的性别推荐相关模板。④嵌套式结构化节点。胸外科电子病历的每一个节点都包含了专科字典，节点嵌套节点，字典嵌套字典。例如，既往史属于大节点，里面嵌套多个数据元小节点。个人使中的" 吸烟史" 在字典选项上提供了" 无吸烟史" 和" 有吸烟史" ，医生选择" 有吸烟史" ，自动生成有吸烟史的详细描述：" 吸烟时长" 、" 吸烟量（支/每年）”、" 戒烟标志" 等。这样有效减少了医生手工书写的内容，提高效率，减少差错。同时，在一定程度上也规范了医生书写病历的标准，毕竟每位医生都有自己的书写病历风格，在这里我们采用了定义好的嵌套字典，有效规避了风格的不统一。⑤多信息节点共享调用。通过结构化的电子病历，将病史、查体、化验检查结果、治疗方法和预后联系在一起，并分析出最科学的临床路径。多个模板使用同一个节点，避免信息重复录入，例如入院记录中主诉、现病史、既往史等信息与首次病程中使用相同的节点，入院记录完善后自动生成首程数据。⑥字典关联片段功能，减少手工录入。电子病历不应该为结构化而结构化，需要医生逐条录入，很不方便。多层次结构化的电子病历更方便医生的录入，例如，描述体检发现的情况，主诉为：体检发现左肺多发肿物。通过下拉菜单，先选择出" 体检发现" ；在子菜单中选择方向" 左/左上/左下/右/右上/右中/右下/前/前上/中/后/后上/后下" ；再相继选择器官" 肺/纵隔/食管/胸壁/肋骨" ，最后选择类型" 结节/包块/肿物/肿瘤" 。通过数据量化标准的模式，把文字录入式的描述降到了最少。这样，操作虽然简单，但得出的病历却很详尽。⑦多信息节点片段。按病种制作相关片段，医生按F10弹出相关片段，根据患者的诊断不同，可选择不同内容的片段进行使用，每个片段中的内容都可以进行结构化。⑧诊断与手术实现结构化、标准化录入。非结构化的病历，诊断录入时只录入诊断名称，而诊断支持结构化录入后，支持录入方位、部位及诊断说明，例如患者的诊断为肺恶性肿瘤，非结构化时医生可能只写" 肺恶性肿瘤" ，诊断结构化、标准化以后，医生可将诊断描述为" 右上肺恶性肿瘤（腺癌）" ；手术结构化也是一样的作用，原本为" 胸腔镜下肺癌根治术" ，支持结构化录入以后，医生可以对手术名称进行补充说明为" 胸腔镜下肺癌根治术（右上肺）" ，使手术名称更加详细。

## 8 讨论

### 8.1 系统应用评价

胸外科结构化、标准化电子病历探索出一种病历记录要素与数据库联动专科电子病历新模式，有利于：①提高临床工作效率，减轻劳动负荷。经过对先后应用结构化、标准化电子病历和Word文档病历的医师书写病历时间比较后，一份完整的大病历的书写时间由原来的30 min缩短到10 min左右。②利用标准化、结构化的高质量数据实施医疗过程的科学管理和智能化控制，提高临床医疗质量，使胸外科的信息化向智慧医疗发展。③在研究中发现采用结构化、标准化后，医生的快速录入文字错误率、病历漏项率也明显降低，准确收集临床资料，存储在CDR，随时可调用分析，为开展临床科研中大量的病历资料进行科学的收集和深入的挖掘提供有效资源，为大数据平台建设奠定基础。

### 8.2 未来展望

厦门大学附属第一医院胸外科结构化、标准化专科电子病历的应用，为信息共享和专科电子病历发展提供了参考，但也对未来如何复制推广，使患者的病历记录能够跨区域交流，为远程会诊提供更细更完整的资料，使患者的胸外科疾病诊治也能实现" 全球通" ，以及实现在大量高质量信息数据基础上的胸外科大数据平台建设也提出新的思考和挑战。
